# Results of 1‑year follow‑up after umbilical hernia with rectus abdominis muscle diastasis repair using endoscopic subcutaneous onlay approach (SCOLA)

**DOI:** 10.20452/wiitm.2024.17889

**Published:** 2024-07-31

**Authors:** Mindaugas Kiudelis, Matas Pažusis, Linas Venclauskas, Eglė Kubiliūtė, Algirda Venclauskienė

**Affiliations:** Department of Surgery, Medical Academy, Lithuanian University of Health Science, Kaunas,, Lithuania; Department of Radiology, Medical Academy, Lithuanian University of Health Science, Kaunas,, Lithuania

**Keywords:** diastasis, hernia, SCOLA, seroma, umbilical

## Abstract

**INTRODUCTION:**

Endoscopic subcutaneous onlay approach (SCOLA) mesh repair in combination with anterior plication of diastasis has recently become a commonly performed procedure.

**AIM:**

The aim of this study was to analyze the results of 1‑year follow‑up in patients after umbilical hernia with rectal abdominal muscle diastasis repair using endoscopic SCOLA.

**MATERIALS AND METHODS:**

Our prospective cohort study included patients who underwent elective surgery for small‑ (<2 cm) and medium‑ (2–4 cm) size primary umbilical hernia with diastasis recti. The follow‑up period was 12 months. Hernia recurrence and postoperative seroma diagnosis were based on the patient’s physical examination and ultrasound assessment. The Carolinas Comfort Scale questionnaire was used to evaluate the quality of life after the surgery.

**RESULT:**

One hundred patients underwent surgery for umbilical hernia with rectal abdominal muscle diastasis repair. Their mean (SD) age was 39.6 (11.8) years. Most of the patients (n = 77) were women. We found no hernia or diastasis recurrence during 1‑year follow‑up. Seroma was found in 15 patients during the first month of follow‑up. The rate of seroma was 11% after 3 months and 2% after 6 months of follow‑up. Almost all the patients reported mild or moderate symptoms during daily activities after the surgery.

**CONCLUSION:**

SCOLA is a safe and effective technique for patients with small umbilical hernia with diastasis. It provides an acceptable cosmetic result for carefully selected patients, low postoperative pain, and good quality of life.

## INTRODUCTION 

Umbilical or epigastric hernias account for 6% to 14% of all abdominal wall hernias in adults.^1^^;^^2^^;^^3^^;^^4^ Diastasis of the rectus abdominis (DRA) muscle is a common condition with functional and cosmetic issues that can occur in both sexes, with a prevalence of 30%–70%.^5^ DRA is defined as distancing from the muscular borders in the midline above 2.2 cm. It is more common in women after pregnancy, and can be associated with umbilical hernias.^6^^;^^7^^;^^8^^;^^9^^;^^10^ Diastasis is usually an esthetic problem manifested as a bulging in the anterior wall of the abdomen. Sometimes it can become a symptomatic problem with low back pain, digestive disorders (constipation), pelvic floor muscle alteration, or a urogynecologic pathology. These esthetic and symptomatic problems usually affect the patient quality of life. ^7^

According to the European Hernia Society (EHS), diastasis can be classified by the degree of rectus abdominis muscles separation as D1 (2–3 cm), D2 (3–5 cm), and D3 (>5 cm)Table 1. A new EHS classification included 2 types of patients: T1 after pregnancy (with [H1] or without [H0] concomitant umbilical or epigastric hernia) and T2 with adiposity (with [H1] or without [H0] concomitant umbilical or epigastric hernia).^11^ At present, controversy exists over surgical indications for DRA repair or surgical technique.^12^ The most accepted indications for surgery are symptoms of DRA, such as esthetic deterioration: a lump (bulging), more evident in multiparous women, and DRA associated with symptomatic umbilical or epigastric hernia.^11^

**Table 1:** European Hernia Society diastasis recti classification

T (type)	D (inter‑rectus distance)	H (concomitant umbilical and / or epigastric hernia)
T1 after pregnancy	D1 >2–3 cm	H0 without
	D2 >3–5 cm	
T2 with adiposity	D3 >5 cm	H1 presen

In recent years, several minimally‑invasive surgical techniques for DRA diastasis with abdominal wall hernias repair have been described.^5^^;^^6^^;^^7^^;^^8^^;^^9^^;^^10^^;^^12^ They included endoscopic subcutaneous onlay approach (SCOLA) mesh repair in combination with anterior plication of DRA,^9^ but in some cases the technique was named preaponeurotic repair of diastasis recti or endoscopic–assisted linea alba reconstruction. Endoscopic SCOLA has recently become a commonly performed procedure. Its concept involves abdominoplasty with dissection of subcutaneous tissue from the lower abdomen to the costal margin and plication of the DRA at the midline, with mesh placement in most cases, performed with small incisions and without any excess skin removal. Early results show good outcomes of this approach. Seroma formation is the main complication after endoscopic DRA repair. The average rate of seroma formation after SCOLA varies from 5% to 40%. ^10^

### AIM 

The purpose of this study was to analyze the results of 1‑year follow‑up in the patients after umbilical hernia with DRA repair using the new surgical technique of SCOLA.

## MATERIALS AND METHODS 

The study protocol was evaluated and approved by the Ethics Committee of the Lithuanian University of Health Sciences (BEC‑MF‑04). The study was registered in the International Standard Randomised Controlled Trial Number registry (IS‑ RCTN28583690). The study was a prospective cohort one, and included patients who underwent elective surgery for small‑and medium‑size primary umbilical hernia with DRA. The patients were operated in the University Hospital, Department of Surgery, between January 1, 2019 and December 31, 2022.

All patients were examined in our outpatient department 1, 3, 6, and 12 months after the surgery. Hernia / diastasis recti recurrence (interrectus distance >2cm) and postoperative seroma diagnosis were based on the physical and ultrasound examination performed by an experienced radiologist. The patient’s age, sex, hospital stay duration, hernia size, postoperative general and wound‑related complications, recurrence rate, postoperative pain, and use of analgesics were analyzed. Postoperative pain was evaluated with Visual Analogue Scale (VAS).

The Carolinas Comfort Scale (CCS) questionnaire was used to evaluate the quality of life after the procedure. The score of 0 means no symptoms, of 1 mild but not bothersome symptoms, 2 mild and bothersome symptoms but not daily, 3 moderate and / or daily symptoms, 4 severe symptoms, and 5 disabling symptoms. All the patients were asked to fill the questionnaire 1 week and 1 month after the surgery.

Umbilical hernias were divided into 3 groups based on their size according to the EHS recommendations:^13^ small (<2 cm), medium (2–4cm), and large (>4 cm). Diastasis recti was classified according to the new EHS recommendations^11^ based on separation between inter‑rectus distance as: D1, D2, and D3, as well as T1, T2, H0, or H1. ^11^

### Surgical technique 

Indications for diastasis plication without mesh repair were female sex, age below 30 years, body mass index (BMI) below 25 kg/m^2^, hernia defect size below 2 cm, and diastasis recti size below 3 cm.

Indications for diastasis recti plication and mesh placement included hernia defect size between 2 and 6 cm and / or diastasis recti size between 3 and 6 cm.

We did not perform the SCOLA procedure in patients with DRA above 6 cm or with hernia greater than 6 cm due to excessive skin amount left after the surgery. All the patients were operated by experienced surgeons (MK, LV).

All the patients received antibiotic prophylaxis with 2 g of cefazolin at the induction of anesthesia. We used 3 trocars (one 12 mm‑long for camera and two 5 mm‑long working trocars) placed in the suprapubic region. Pre‑aponeurotic dissection was carried out superiorly to the xiphoid process and bilaterally, using harmonic energy. The size of the dissection was 6–8 cm laterally on each side in the cases with out the mesh placement, and 10 to 12 cm when the mesh was used.

Umbilical hernia defect was closed using interrupted nonresorbable sutures. Anterior diastasis plication started at the xiphoid process and we used running, barbed, monofilament, nonresorbable sutures. A light polypropylene mesh was placed where needed and fixated with continuous 3/0 prolene suture reinforcing the mesh edges, and interrupted sutures in the middle of the mesh. The mesh was sized to fit the entirety of the dissected subcutaneous space. The subcutaneous space drainage was provided after the operation. The drain was removed when the secretion was below 50 ml/d. All the patients wore an abdomen corset belt for 1 month after the surgery.

**Table 2:** General patient characteristics

Parameter		Value
Patients, n		100
Age, y		36.6 (11.8)
Sex, n	Men	23
	Women	77
ASA score		2 (1–2)
BMI, kg/m^2 ^		21.6 (2.6)
Hernia occurrence time, mo		13.5 (5.1)
Hernia size, cm, n		
<2		49
2–4		51
Hernia defect area, cm^2^		3.3 (1.6)
Diastasis recti, cm, n		
2–3		35
3–5		65
Diastasis recti width, cm		2.9 (0.4)

Data are presented as mean (SD) or median (interquartile range) unless stated otherwise.

Abbreviations: ASA, American Society of Anesthesiologists; BMI, body mass index

**Table 3:** Early follow‑up results (during hospitalization)

Parameter	Value
Operating time, min	134 (49)
With mesh	130 (36)
Without mesh	135 (53)
Hospital stay, d	2 (1–2)
Postoperative pain, VAS	
3 h	4.97 (1.67)
6 h	4.7 (1.93)
9 h	3.38 (2.2)
24 h	2.47 (1.39)
48 h	2.3 (1.38)
72 h	1.18 (0.68)
96 h	1 (0.51)
Early complications, n	
Hematoma	1

Data are presented as mean (SD) or median (interquartile range) unless stated otherwise.

Abbreviations: VAS, visual analog scale

**Table 4:** Follow ‑up data

Parameter	Value
Seroma, n	
1 mo	15
Early < 2 wks	4
Late > 2 wks	11
3 mo	11
6 mo	2
12 mo	2
Hypoesthesia, n	
6 mo	63
12 mo	17

### Statistical analysis 

Statistical analyses were performed using SPSS Statistics 20.0 for Windows (IBM, Armonk, New York, United States). Data were expressed as mean and standard deviation. A P value below 0.05 was considered significant.

## RESULTS 

One hundred patients underwent elective surgery for umbilical hernia with diastasis recti repair over the period of 2 years. The youngest patient was 18 years old and the oldest 77 years old. Most of the patients (n = 77) were women. All the women gave birth (at least 2 years before the surgery). All the hernias were umbilical ones. Patient general characteristics are summarized in Table 2.

Operating time was about 2 hours. Thirty‑two patients were operated with mesh repair and 68 without the mesh placement. There were no intraoperative complications during the study period. The drains were removed within 2 days after the surgery. The worst pain was reported on the first day after the operation. Only 1 patient had postoperative complications (wound hematoma) during hospital stay, which healed without surgery or other interventions. Mean (interquartile range) hospital stay was 2 (1–2) days. Early follow‑up results are summarized in Table 3.

No patients were lost to follow‑up, and there was no hernia or DRA recurrence for 1‑year follow‑up. Early seromas (<2 weeks after the operation) were detected in 4 patients after DRA repair with mesh placement, while late seromas (>2 weeks after operation) were found in 11 oth‑ er patients. In 3 patients, late seromas were observed after DRA repair and mesh placement, while 8 cases were noted after diastasis plication without mesh repair. There were 11 cases of seroma within 3 months after the surgery: 3 of them after DRA plication and mesh repair and 8 after DRA plication. Only 2 patients had seromas 6 and 12 months after the surgery. Both of them received DRA plication and mesh repair. The late seromas for 8 patients were aspirated under ultrasonographic control (mean [SD] volume of the seroma was 68.8 [21] ml) within the first month of follow‑up.

Sixty‑three patients experienced abdominal skin hypoesthesia 6 months after the surgery, and 17 patients remained hypoesthetic 12 months after the operation. Late follow‑up results are summarized in Table 4 .

All the patients were asked about the esthetic abdominal wall results 1 year after the surgery. Ninety‑nine patients evaluated the esthetic results as perfect or good Table 5 .

None of the patients had excess of skin or subcutaneous tissue after the SCOLA procedure.

Almost all individuals reported mild or moderate symptoms during daily activities after the SCOLA surgery. There were no significant differences between the DRA plication and DRA plication with mesh placement groups, as compared with the CCS scores after the surgery Table 6.

**Table 5:** Patient satisfaction with abdominal wall esthetics 12 months after surgery

Degree of satisfaction	Number of patients
Excellent	93
Good	6
Neutral	1
Poor	0

## DISCUSSION 

DRA is a common condition, especially during and post pregnancy, and it is characterized by thinning and widening of the linea alba combined with sagging of the abdominal wall muscles. In women, DRA with a concurrent hernia and excess lower abdominal skin is defined as postpartum wall insufficiency syndrome (PPAWIS). This syndrome, apart from back pain or pelvic floor organ problems, imposes issues with self‑acceptance, self‑esteem, and sexual life.^14^ Most patients with diastasis are treated conservatively, as DRA is usually not associated with symptoms or risk of complications.^7^ The most widely accepted indications for surgery are symptomatic DRA, esthetic deterioration (a lump or bulging, particularly in multiparous women), and DRA associated with symptomatic umbilical or epigastric hernia. ^11^

Conventional surgery and abdominoplasty are the most often used techniques, which allow for treatment of diastasis and excess skin with subcutaneous fat. For patients who do not have skin excess, various minimally‑invasive techniques have been described as alternative treatments for DRA. Regardless of the form of access, it seems that the plication techniques have greater acceptance in the literature, as opening of the midline is associated with a potentially greater risk of incisional hernia. ElHawary et al^15^ performed a systematic review and pooled analysis of complications and recurrence rates comparing open and laparoscopic management of DRA. They found that total complication rate in patients who underwent open DRA repair with herniorrhaphy was 13.3%, while in patients who underwent laparoscopic repairs it reached 14.5% (P <0.05).

Our study analyzed a fully endoscopic access through 3 suprapubic trocars, using working pressure of C0_2 _of 12–14 mm Hg for the correction of umbilical hernias simultaneously with diastasis recti. Classic SCOLA procedure involves endoscopic DRA plication with only mesh placement. We made some modifications to this procedure, as we did not place the mesh following DRA plication in every patient. According to the EHS guidelines on DRA management,^11^ plication of the linea alba may be sufficient to repair a diastasis associated with small (less than 1 cm) umbilical / epigastric hernias. We did not place the mesh after DRA plication in young women with BMI below 25 kg/m^2^ and small (less than 2 cm) umbilical hernias with diastasis recti size below 3 cm.

Due to a lack of clinical trials, there are no strong recommendations from the EHS regarding the use of mesh in diastasis surgery. The EHS guidelines state that there is no need for DRA plication below 1 cm.^11^ The width of the linea alba up to 1 cm is considered an anatomical norm, and so the use of the mesh is questionable. We did not operate patients who had muscle diastasis up to 2 cm without umbilical hernia, as we considered this to be a physiological norm, but we operated the patients with diastasis and umbilical hernia. Therefore, we chose to use mesh repair if the diastasis exceeded 3 cm and umbilical hernia was greater than 2 cm.

The SCOLA technique and its results were described in several publications.^5^^;^^7^^;^^8^^;^^9^^;^^10^^;^^12^ Most of the patients had umbilical / epigastric hernias with diastasis recti. Postoperative follow‑up varied widely from 2 to 36 months, while recurrence rate was usually low (0%–2%).^5^^;^^7^^;^^8^^;^^9^^;^^10^ Dong et al^9^ reported 18.8% recurrence rate, but their study included only 16 patients. We noted no recurrences during 1‑year follow‑up, which is similar to other literature reports.^5^^;^^7^^;^^8^^;^^9^^;^^10^^;^^12^ On the other hand, according to the literature, for diastasis recti greater than 6–7 cm or associated with severe musculoaponeurotic laxity, simple plication would not be sufficient to achieve correction and good long‑term results. In these cases, the use of reinforced prosthesis would be recommended. We did not perform the SCOLA procedure in patients with DRA greater than 6 cm.

Late seroma was the most frequent postoperative complication (11%), as in a majority of publications on endoscopic techniques (3.8%–62%), being no different from the complication rate after conventional abdominoplasty.^8^ The seromas were usually asymptomatic. We found 2 cases of seroma 6 and 12 months after the SCOLA procedure. Numerous efforts have been made to reduce the seroma formation, including the use of drains, abdominal binders, and intraoperative fibrin sealant, or limiting the extent of lateral subcutaneous dissection during the operation. Some authors^9^ recommend keeping subcutaneous drain for 10–14 days to lower the risk of seroma formation. We usually removed the drains on the second postoperative day, when the amount of secretion was below 50 ml/d, but seromas appeared later on. Seromas are most often evidenced 20–50 days postoperatively, but they usually reabsorb spontaneously by day 65.^5^^;^^7^^;^^8^^;^^9^^;^^10^^;^^12^

**Table 6:** Carolinas Comfort Scale scores (after surgery)

Parameter		1 Week			1 Month	
Domain	DRA plication + mesh placement (n = 32)	DRA plication without mesh (n = 68)	*P *value	DRA plication + mesh placement (n = 32)	DRA plication without mesh (n = 68)	*P *value
Lying down	1.7 (0.81)	1.62 (0.72)	>0.05	0.4 (0.24)	0.41 (0.33)	>0.05
Bending over	2.78 (0.2)	2.8 (0.9)	>0.05	1.2 (1)	1.09 (1)	>0.05
Sitting up	1.3 (0.98)	1.27 (0.96)	>0.05	0.6 (0.3)	0.66 (0.31)	>0.05
Activities of daily living	1.6 (1.03)	1.62 (1.01)	>0.05	1.11 (0.65)	1.1 (0.63)	>0.05
Coughing or deep breathing	2.1 (1.15)	1.89 (1.1)	>0.05	0.9 (0.41)	0.85 (0.37)	>0.05
Walking	1.55 (0.83)	1.54 (0.82)	>0.05	0.74 (0.23)	0.71 (0.2)	>0.05
Walking up the stairs	1.9 (1.12)	1.89 (1.07)	>0.05	0.9 (0.19)	0.8 (0.23)	>0.05
Exercising	3.94 (1.05)	1.94 (1.02)	>0.05	2.9 (1.1)	2.1 (1.15)	>0.05
Total	30.15 (9.33)	28.12 (11.67)	>0.05	8.97 (3.15)	7.68 (4.21)	>0.05

Data are presented as mean (SD).

Abbreviations: DRA, diastasis of the rectus abdominis

In our opinion, the formation of seromas was greatly influenced by too wide separation of the subcutaneous tissue from the aponeurosis, especially when placing the mesh. At the end of the operation, the dissected subcutaneous tissue was not fixed (as it would usually be done during an open operation) to the aponeurosis, because technically it is a very complicated and long‑lasting, uncomfortable stage. There are no clear recommendations or common opinions regarding the use of vacuum drains for this operation and how long to maintain the drains. Some clinical studies have not shown any advantages of using vacuum drains in the prevention of seromas during open surgery. The use of a glue also did not show superiority in the prevention of seroma.^16^ A few clinical studies from Poland and Israel used hypertonic solution at the end of the operation for seroma prevention.^17^^;^^18^ A pilot cohort study of Zamkowski et al^18^ compared the effect of a hypertonic solution for prevention of seromas in patients who underwent open elective abdominal wall reconstruction surgery and patients with PPAWI. In the short‑term, they demonstrated that intraoperative hypertonic saline irrigation significantly decreased the amount of the drained fluid and shortened the hospital stay. In their prospective study, Dudai et al^17^ used a hypertonic solution at the end of the operation for seroma prevention in patients who underwent SCOLA procedure for ventral hernia. The outcomes were acceptable, but a single clinical study is not enough to draw generalized conclusions. Both clinical studies^17^^;^^18^ demonstrated that using intraoperative hypertonic saline irrigation can significantly reduce postoperative seroma formation, but more multicenter studies in larger groups of patients are needed to form appropriate recommendations or conclusions.

In American experience, seroma was more common in patients with higher BMI.^9^ Our study demonstrated that 99% of the patients assessed the cosmetic results of the operation as excellent and very good 1 year after the surgery. In Brazil, 93.7% patients reported being satisfied with the outcome of the SCOLA procedure. ^7^

Our study showed that hypoesthesia was present in 63% of the patients 6 months after the SCOLA procedure, and remained in 17% of them 1 year after the surgery. According to Muas et al,^5^ hypoesthesia occurs in 100% of patients immediately after the operation, but total recovery of sensitivity is observed between 2 and 6 months after surgery, without any sequels. In our study, 17% of patients still had hypoesthesia 1 year after the surgery, and such results are difficult to explain.

We analyzed the quality of life after the SCOLA surgery using the CCS and comparing the patients with DRA plication with and without mesh placement. Our hypothesis was that DRA plication with mesh placement worsens the quality of life as compared with DRA plication alone. We found that mesh placement after DRA plication had no significant effect on all quality of life areas 1 week and 1 month after the surgery, and quality of life was rated good enough 1 month after the SCOLA procedure Table 6.

## CONCLUSIONS 

SCOLA is a safe and effective technique for patients with small umbilical hernia and diastasis recti. This technique gives an acceptable cosmetic result for carefully selected patients, and is associated with low postoperative pain, short hospital stay, and good quality of life. 

Long‑term results showed no hernia and diastasis recurrence after 1‑year follow‑up. The main problem after SCOLA was seroma (11% 3 months after the surgery).
